# VISTA: a classifier for metagenomic subspecies and community state typing of the vaginal microbiome

**DOI:** 10.1128/mra.00612-26

**Published:** 2026-09-11

**Authors:** Johanna B. Holm, Amaury Maros, Amanda Williams, Michael France, Jacques Ravel

**Affiliations:** 1 Center for Advanced Microbiome Research and Innovation, University of Maryland Baltimore School of Medicine 12264, Baltimore, Maryland, USA; 2 Institute for Genome Sciences, University of Maryland School of Medicine 12264, Baltimore, Maryland, USA; 3 Department of Microbiology and Immunology, University of Maryland Baltimore School of Medicine 12264, Baltimore, Maryland, USA; University of Michigan, Ann Arbor, Michigan, USA

**Keywords:** human microbiome, metagenome, vaginal microbiota, host-microbe, bioinformatics, genomics, species, subspecies, strain, function

## Abstract

Metagenomic community state types (mgCSTs) capture within-species genetic and functional diversity and community structure of the vaginal microbiome, enabling precise links between microbiome composition, function, and health-related risk. VISTA, the Vaginal Inference of Subspecies and Typing Algorithm, is a two-step classifier that assigns mgCSTs to vaginal metagenomes, providing standardized, scalable classifications.

## ANNOUNCEMENT

Using metataxonomic data, vaginal microbiota can be categorized into five community state types (CSTs), each predominated by specific species (CST I: *Lactobacillus crispatus*; CST II: *Lactobacillus gasseri*; CST III: *Lactobacillus iners*; CST V: *Lactobacillus jensenii*) or characterized by a paucity of *Lactobacillus* and a diverse array of anaerobes (CST IV) (1, 2). CSTs enable dimensionality reduction of complex microbiota data sets to facilitate evaluation of associations between the vaginal microbiota and urogenital and reproductive health (2–14). Metagenomic sequencing of the vaginal microbiome has revealed substantial genetic and functional diversity, encompassing more than 1.7 million non-redundant genes (15). Accounting for this diversity allows for more precise risk estimates and helps elucidate the mechanisms linking the microbiome to clinical outcomes.

**Fig 1:**
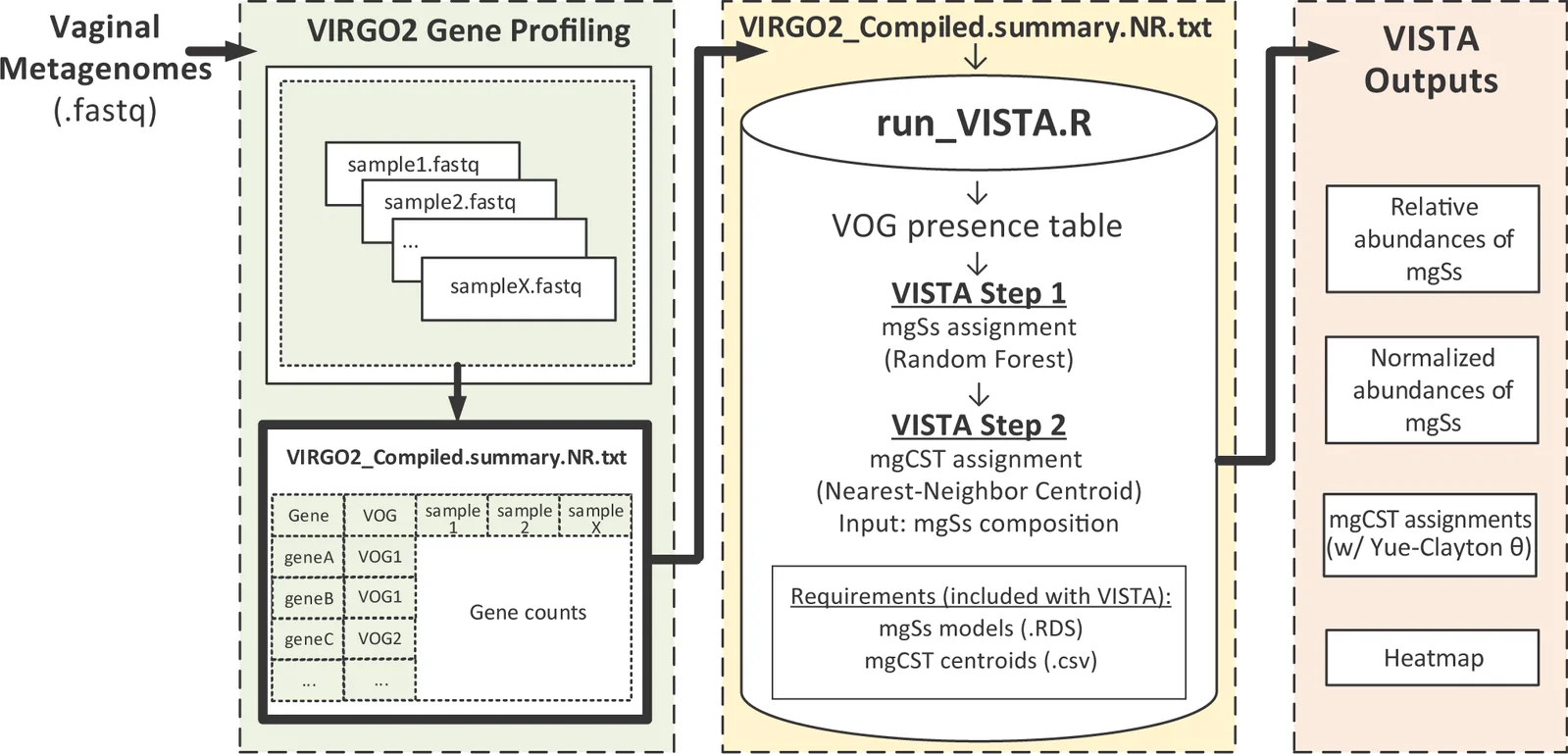
Overview of the VISTA workflow. Metagenomic sequencing reads are first processed with VIRGO2 to generate a compiled gene abundance profile. VISTA then performs a two-step classification procedure: (i) assignment of mgSs using species-specific random forest models and (ii) assignment of mgCSTs using nearest-centroid classification based on inferred mgSs composition. Outputs include mgSs abundance matrices, mgCST assignments, similarity scores, and visualization files.

To facilitate such analyses and promote cross-study comparability, we developed VISTA, the Vaginal Inference of Subspecies and Typing Algorithm. VISTA is a two-step classifier that first assigns metagenomic subspecies (mgSs) using species-specific gene presence data and then assigns metagenomic community state types (mgCSTs) based on mgSs and species composition (16) (Fig. 1). The input consists of gene profiles derived from metagenomes mapped to VIRGO2, a comprehensive gene catalog of the vaginal microbiome (15). The outputs are (i) a sample-by-mgSs abundance matrix and (ii) per-sample mgCST classifications. The biological utility of these classifications is described in a companion manuscript (16).

To generate the VISTA reference framework, we used VIRGO2-v.1.0.0-mapped gene abundance profiles from 2,528 vaginal metagenomes as input (15). Gene counts were multiplied by the sequencing read length and divided by gene length to estimate gene coverage. A vaginal orthologous group (VOG) (15) presence table was then generated, wherein a VOG was considered present if at least one of its member genes exhibited ≥0.5× coverage. Species were eligible for mgSs analysis if ≥80% of samples containing the species achieved ≥90% coverage of its average coding sequence repertoire, resulting in 73 eligible species. For each species, samples were clustered based on Jaccard distances calculated from binary VOG profiles using Ward’s minimum variance hierarchical clustering implemented in vegan-v.2.6-8 under R-v.4.4.0 (17–19). Initial clusters were identified using dynamicTreeCut-v.1.63-1 (20). Cluster robustness was evaluated by comparison of observed silhouette scores with those derived from 10 randomized presence-absence matrices in which 10% of entries were randomly perturbed. Clusters were iteratively merged until observed silhouette scores exceeded those from the randomized data. This process defined 262 mgSs across the 73 species. Samples were subsequently represented by the relative abundances of the constituent mgSs, and mgCSTs were identified by clustering samples using Jensen-Shannon distances and Ward’s minimum variance method. This approach defined 25 mgCSTs that serve as the reference classifications used by VISTA. Default parameters were used except where otherwise noted. Tenfold cross-validation yielded a mean out-of-bag error rate of 8.9% for mgSs assignment and a mean accuracy of 87.8% for mgCST classification.

Using these reference classifications, VISTA implements a two-step classification workflow with R. First, random forest models (randomForestSRC-v.3.3.1) assign mgSs from VIRGO2-derived, species-specific gene presence profiles (21, 22). Second, a nearest-neighbor centroid classifier assigns samples to one of the 25 mgCSTs based on their predicted mgSs composition. While not all microbiome niches exhibit discrete community types, the VISTA framework can be applied to other microbial ecosystems, supporting population-level epidemiological investigations.

## Data Availability

VISTA v2.0 is available at https://github.com/JHolm-Lab/VISTA (DOI: 10.5281/zenodo.21937773) under the GNU General Public License version 3 (GPL-3.0). The software is implemented in R and has been tested on Linux- and macOS-based systems.

## EDITOR'S NOTE

**Ed. Note**: This article was handled by Pat Schloss, who acted as a guest editor in consultation with the editor in chief, Irene Newton. Dr. Schloss's role as the guest editor of this article is outside of his duties as the chair of the Journals Committee.
